# The diagnostic accuracy for ARDS of global versus regional lung ultrasound scores - a post hoc analysis of an observational study in invasively ventilated ICU patients

**DOI:** 10.1186/s40635-019-0241-6

**Published:** 2019-07-25

**Authors:** Luigi Pisani, Veronica Vercesi, Patricia S. I. van Tongeren, Wim K. Lagrand, Stije J. Leopold, Mischa A. M. Huson, Patricia C. Henwood, Andrew Walden, Marry R. Smit, Elisabeth D. Riviello, Paolo Pelosi, Arjen M. Dondorp, Marcus J. Schultz, for the Lung Ultrasound Consortium

**Affiliations:** 1 Department of Intensive Care, Amsterdam University Medical Centers, AMC, Meibergdreef 9, 1105 AZ Amsterdam, The Netherlands; 2 Mahidol–Oxford Tropical Medicine Research Unit (MORU), Mahidol University, Bangkok, 10400 Thailand; 3 Department of Surgical Sciences and Integrated Diagnostics, San Martino Policlinico Hospital, IRCCS for Oncology, University of Genoa, 16132 Genoa, Italy; 4 Department of Internal Medicine, Tergooi Hospital, 1261 AN Blaricum, The Netherlands; 5 Department of Internal Medicine, Amsterdam University Medical Centers, AMC, 1105 AZ Amsterdam, The Netherlands; 6 Division of Pulmonary, Critical Care and Sleep Medicine, Beth Israel Deaconess Medical Center and Harvard Medical School, Boston, MA 02215 USA; 7 Department of Emergency Medicine, Brigham and Women’s Hospital, Boston, MA 02115 USA; 8 Department of Intensive Care, Royal Berkshire Hospital, Reading, RG1 5LE UK; 9 Laboratory of Experimental Intensive Care and Anesthesiology (L·E·I·C·A), Amsterdam University Medical Centers, AMC, 1105AZ Amsterdam, The Netherlands

**Keywords:** ARDS, Lung ultrasound, Diagnosis, Diagnostic accuracy, Ventilation, Intensive care

## Abstract

**Background:**

Semi-quantification of lung aeration by ultrasound helps to assess presence and extent of pulmonary pathologies, including the acute respiratory distress syndrome (ARDS). It is uncertain which lung regions add most to the diagnostic accuracy for ARDS of the frequently used global lung ultrasound (LUS) score. We aimed to compare the diagnostic accuracy of the global versus those of regional LUS scores in invasively ventilated intensive care unit patients.

**Methods:**

This was a post-hoc analysis of a single-center observational study in the mixed medical–surgical intensive care unit of a university-affiliated hospital in the Netherlands. Consecutive patients, aged ≥ 18 years, and are expected to receive invasive ventilation for > 24 h underwent a LUS examination within the first 2 days of ventilation. The Berlin Definition was used to diagnose ARDS, and to classify ARDS severity. From the 12-region LUS examinations, the global score (minimum 0 to maximum 36) and 3 regional scores (the ‘anterior,’ ‘lateral,’ and ‘posterior’ score, minimum 0 to maximum 12) were computed. The area under the receiver operating characteristic (AUROC) curve was calculated and the best cutoff for ARDS discrimination was determined for all scores.

**Results:**

The study enrolled 152 patients; 35 patients had ARDS. The global score was higher in patients with ARDS compared to patients without ARDS (median 19 [15–23] vs. 5 [3–9]; *P* < 0.001). The posterior score was the main contributor to the global score, and was the only score that increased significantly with ARDS severity. However, the posterior score performed worse than the global score in diagnosing ARDS, and it had a positive predictive value of only 50 (41–59)% when using the optimal cutoff. The combined anterolateral score performed as good as the global score (AUROC of 0.91 [0.85–0.97] vs. 0.91 [0.86–0.95]).

**Conclusions:**

While the posterior score increases with ARDS severity, its diagnostic accuracy for ARDS is hampered due to an unfavorable signal-to-noise ratio. An 8-region ‘anterolateral’ score performs as well as the global score and may prove useful to exclude ARDS in invasively ventilated ICU patients.

## Background

Point-of-care ultrasound is an increasingly used clinical imaging modality for diagnostic and monitoring purposes in a number of common intensive care unit (ICU) conditions, including the acute respiratory distress syndrome (ARDS) [1, 2]. Lung ultrasound (LUS) is an attractive alternative to chest radiography or CT scan [3], in particular in places where these latter imaging modalities are scarce or absent [4]. By now, several approaches integrate LUS in the diagnosis and monitoring of ARDS [5–8]. The recently proposed Kigali modification of the Berlin Definition for ARDS is a pragmatic attempt to replace chest radiography or computer tomography in the diagnostic process of ARDS [6]. Its excellent sensitivity for ARDS was recently confirmed [9].

One challenge with LUS is the way to report and interpret its findings, which are peculiar being a mix of non-numeric artifactual and real sonographic images [10–13]. One frequently used approach is semi-quantification of lung aeration across 12 lung regions into a numerical score [14], of which the steep learning curve was recently demonstrated [15]. The so-called ‘global score,’ a lung aeration score that correlates well with CT-quantified aeration [16], is in fact nothing more than a composite of scores integrating anterior, lateral, and posterior lung regions. All abnormalities in each region count equally within the final score, even while the relative importance and meaning of these abnormalities may be very different. Specifically, the signal-to-noise ratio could be low in those regions that are subject to the development of abnormalities other than ARDS, which is particularly true for posterior regions where compressive and perioperative atelectasis, pre-existing or new infiltrates, and hydrostatic pulmonary edema may concentrate due to a positional gradient [11, 17–20].

We hypothesized that the diagnostic accuracy for ARDS and ARDS severity of the ‘posterior’ score would be inferior to that of the global score, and to that of the ‘lateral’ and ‘anterior’ scores. To test this hypothesis, we determined and compared the diagnostic accuracy of the global score and the three regional scores in a cohort of invasively ventilated ICU patients, on which we reported previously [9]. In addition, we tested whether omitting the posterior score from the global score, i.e., using an ‘anterolateral’ score, would yield a diagnostic accuracy for ARDS comparable to that of the global score.

## Methods

This was a post-hoc analysis of a single-center observational study performed from November 2016 to June 2017 in the mixed medical–surgical ICU of the Amsterdam University Medical Centers, in Amsterdam, The Netherlands. The study was approved by the Institutional Review Board of the Academic Medical Center (approval W17_353 #17.411). The need for written informed consent was waived seen the observational nature of the study.

### Inclusion and exclusion criteria

The original study had the following two inclusion criteria: age ≥ 18 years, and expected to receive invasive ventilation for > 24 h [9]. Exclusion criteria were no LUS examination, performed as part of standard care, in the first 48 h of ventilation, unreliable oximetry data, and not having a chest radiograph or computed tomography scan of the lungs while on at least 5 cm H_2_O positive end-expiratory pressure (PEEP), mandatory to make the diagnosis of ARDS according to the Berlin Definition for ARDS [21]. This post-hoc analysis did not use additional exclusion criteria. The presence of ARDS was assessed by a panel of two clinicians, using the Berlin Definition for ARDS [22] that includes new or worsening respiratory symptoms within 1 week of a known medical clinical insult, a PaO_2_/FiO_2_ < 300 mmHg; bilateral opacities on the chest film or computed tomography (CT) exam, not explained by effusions, collapse, or nodules; and respiratory failure not fully explained by cardiac failure or fluid overload. In case of persistent disagreement on radiographic criteria, radiography results were discussed with a third clinician who had no access to other clinical information, to reach consensus.

### Lung ultrasound

A trained and experienced intensivist (VV), not involved in direct patient care and unaware of clinical information or ARDS status, performed the LUS examination using a LOGIQe ultrasound machine (GE Healthcare, Little Chalfont, UK). A convex 2–5 MHz transducer was used with the probe applied longitudinally and perpendicularly to the thoracic wall. LUS consisted of a scan of 12 different regions—6 per hemithorax, i.e., two anterior, two lateral, and two posterior thoracic regions were delimitated as described before [23]. Each region was scored, as follows: ‘0’, A-pattern with ≤ 2 B-lines; ‘1,’ more than two separated B-lines; ‘2’, multiple coalescent B-lines; or ‘3’, lung consolidation.

### Global and regional scores

The global score was calculated by summing the scores of all 12 lung regions, which thus could range from 0 (i.e., normal aeration in all regions) to 36 (i.e., the extreme situation in which all regions had consolidations). Regional scores were calculated by summing the field scores of anterior, lateral, or posterior regions, respectively (range from 0 to 12). An adjusted composite score, called the ‘anterolateral’ score, was derived by summing the anterior and lateral regional scores (range from 0 to 24) [10].

Missing scores values from one or more regions that were non-examinable were complemented by the proportional quotation from the same examination using the formula (final score = actual score × (N° of potential regions / N° of actual regions scanned), where the number of potential regions was 12 or 4 for the global and regional scores, respectively.

### Primary clinical endpoint

The clinical endpoint was ARDS according to the Berlin Definition [21]. The same definition was used to classify ARDS severity as mild, moderate, or severe.

### Statistical analysis

Demographic, clinical, and outcome variables were presented as percentages for categorical variables and as medians with interquartile ranges (IQR) for continuous variables.

The Mann–Whitney *U* test was used to compare LUS scores between patients with and without ARDS, and Kruskal–Wallis statistics to seek significant differences across patients with mild, moderate, and severe ARDS. Pairwise comparisons across groups were explored using the Dunn test with a Benjamini–Hochberg correction for multiple comparisons.

Receiver operating characteristic (ROC) curves for ARDS were drafted for the global score, and the regional scores. The area under the receiver operating characteristic curves (AUROC) with 95% confidence intervals where calculated to determine the diagnostic accuracy for ARDS of global and regional scores. AUROCs were compared using the De Long test [24]. The optimal cutoffs were determined as the highest Youden’s index (sensitivity + specificity − 1) [25]. Sensitivity, specificity, and positive and negative predictive values were calculated, based on these cutoffs.

The diagnostic accuracy based on ROCs was predefined as ‘excellent’ if the AUROC was between 0.9 and 1, ‘good’ between 0.8 and 0.9, ‘moderate’ between 0.7 and 0.8, poor between 0.6 and 0.7, and ‘fail’ when below 0.6. As LUS is a first line diagnostic technique, a positive predictive value below 50% and a negative predictive value below 80% were considered clinically irrelevant [26].

All statistical analyses were performed in R (version 3.3.1, www.r-project.org) and graphs built using GraphPad Prism (version 7.03, www.graphpad.com). A *P* value below 0.05 was considered significant.

## Results

### Patients

**Fig. 1:**
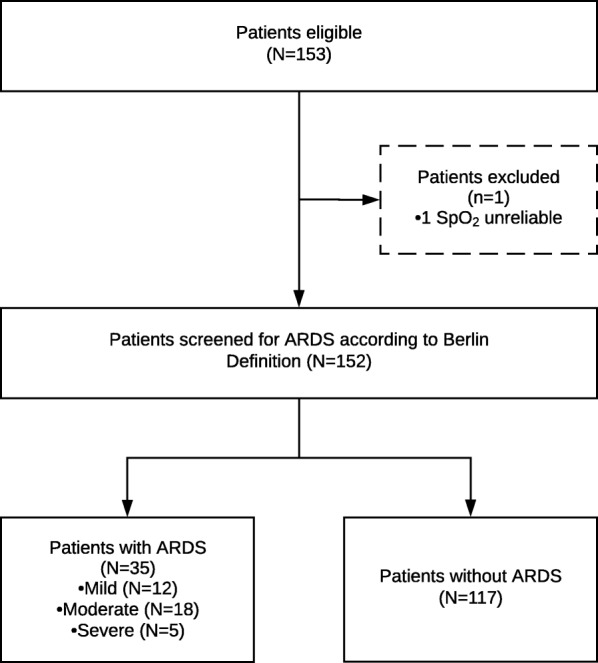
Flow of the patients in this study

Patient flow is shown in Fig. 1, and patient demographic and clinical characteristics are presented in Table 1.

**Table 1:** Patients baseline, ventilation, and outcome characteristics

	No ARDS	ARDS	*P* value	ARDS severity groups			
				Mild	Moderate	Severe	*P* value
*N*	117	35		12	18	5	
Demographics							
Age—years	62 (51, 72)	58 (48, 69)	0.15	66 (52, 70)	57 (29, 69)	53 (46, 55)	0.201
Male—%	69 (59.0)	21 (60.0)	1	5 (41.7)	12 (66.7)	4 (80.0)	0.281
APACHE II	21 (17, 27)	20 (15, 26)	0.324	20 (16, 27)	20 (14, 24)	24 (20, 27)	0.708
APS	38 (15, 52)	39 (16, 55)	0.712	22 (15, 50)	27 (17, 54)	60 (54, 63)	0.333
SOFA	9 (7, 12)	11 (8, 13)	0.218	9 (7, 12)	11 (8, 13)	13 (12, 15)	0.043
Clinical insult^a^							
Sepsis—%	27 (23.1)	11 (31.4)	0.374	4 (33.3)	4 (22.2)	3 (60.0)	0.334
Pneumonia—%	17 (14.5)	17 (48.6)	< 0.001	7 (58.3)	9 (50.0)	1 (20.0)	0.426
Stroke—%	26 (22.2)	1 (2.9)	0.006	1 (8.3)	0 (0.0)	0 (0.0)	0.486
Surgery—%	24 (20.5)	4 (11.4)	0.321	1 (8.3)	2 (11.1)	1 (20.0)	0.620
Trauma—%	15 (12.8)	2 (5.7)	0.362	1 (8.3)	0 (0.0)	1 (20.0)	0.118
Other—%	26 (22.2)	9 (25.7)	0.653	2 (16.7)	7 (38.9)	0 (0.0)	0.210
Ventilation							
PEEP—cm H2O	5 (5, 7)	10 (7, 12)	< 0.001	7 (6, 10)	10 (8, 14)	15 (10, 15)	0.014
PaO_2_/FiO_2_	315 (234, 380)	163 (124, 247)	< 0.001	255 (247, 278)	144 (125, 171)	73 (68, 81)	< 0.001
Pmax—mmHg	17 (14, 22)	28 (21, 31)	< 0.001	26 (22, 29)	29 (20, 33)	29 (27, 30)	0.446
RR—breaths per min	18 (14, 23)	27 (19, 32)	< 0.001	25 (21, 31)	29 (19, 33)	22 (12, 29)	0.692
FiO_2_	0.30 (0.25, 0.40)	0.45 (0.34, 0.60)	< 0.001	0.30 (0.29, 0.33)	0.55 (0.42, 0.60)	1 (0.90, 1)	< 0.001
Minute volume—l/min	8.8 (7.2, 10.8)	10.8 (9.2, 13.3)	< 0.001	9.7 (9.2, 10.6)	12.5 (10.5, 13.3)	14.1 (6.9, 15.2)	0.233
*T*_V_—ml/PBW	8.0 (6.6, 9.2)	6.9 (5.7, 9.3)	0.275	8.6 (6.6, 9.3)	6.9 (5.5, 9.4)	5.9 (3.5, 7.2)	0.279
Outcomes							
Duration of MV—days	3 (1, 8)	9 (3, 18)	< 0.001	4 (2, 11)	12 (7, 18)	3 (2, 19)	0.131
ICU mortality—%	37 (31.6)	13 (37.1)	0.545	3 (25.0)	5 (27.8)	5 (100.0)	0.007
Hospital Mortality—%	47 (40.2)	13 (37.1)	0.845	3 (25.0)	5 (27.8)	5 (100.0)	0.007
Ventilator free days	19 (0, 24)	5 (0, 19)	0.038	16 (2, 21)	7 (0, 18)	0 (0, 0)	0.030

Values are presented as *N*(%) or median (interquartile range)

Ventilatory parameters refer to the moment of the lung ultrasound examination

*APACHE* Acute Physiology and Chronic Health Evaluation score, *APS* acute physiology score, *SOFA* sequential organ failure assessment score, *ARDS* acute respiratory distress syndrome, *ICU* intensive care unit, *FiO*_*2*_ fraction of inspired oxygen, *PEEP* positive end-expiratory pressure, *Pmax* maximal inspiratory airway pressure, *RR* respiratory rate, *TV* tidal volume, *PBW* predicted body weight, *SpO*_2_ pulse oximetry oxygen saturation, *PaO*_2_ arterial partial pressure of oxygen

^a^Non-exclusive categories

Out of 152 patients, 35 (23.0%) had ARDS according to the Berlin Definition for ARDS. Twelve patients were classified as having mild ARDS, and 18 and 5 patients as having moderate or severe ARDS, respectively.

Twelve patients had at least 1 lung region that could not be scored, due to large surgical or drainage dressings, chest tubes, or due to patient positioning, resulting in a total of 36/1824 (2.0%) non-scannable regions.

### Global and regional scores

**Fig. 2:**
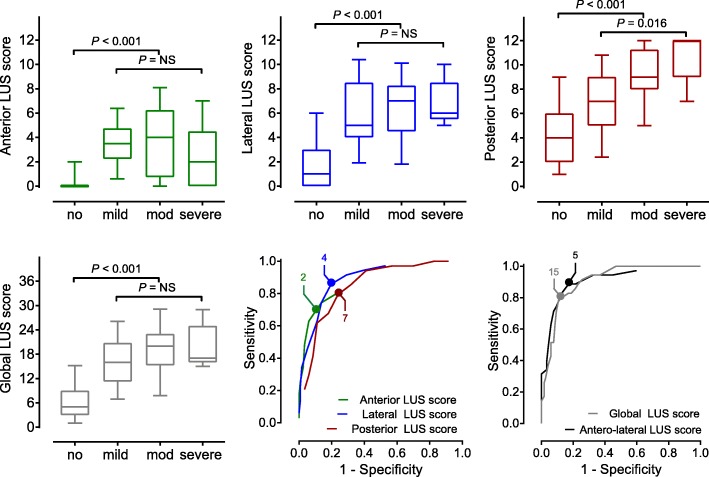
Anterior, lateral, posterior, and global scores and the receiver operating characteristics curves (ROCs) for the diagnostic accuracy for the acute respiratory distress syndrome of the regional and composite scores. Boxes present interquartile ranges while whiskers show 10 to 90 percentiles. Dots in the ROCs represent the best cutoff, and used to calculate sensitivity, specificity, positive and negative predictive value. *LUS* lung ultrasound, *ROC* receiver operating characteristics curve

Global and regional LUS scores and ROC curves are presented in Fig. 2. AUROC, sensitivity, specificity, and negative and positive predictive values are presented in Table 2. Comparisons between the AUROC using the De Long test are shown in Table 3.

**Table 2:** AUROC and ROC-derived cutoffs for ARDS and their diagnostic accuracy measures

Score	AUROC	Optimal cutoff	Sensitivity	Specificity	PPV	NPV
Composite						
Global	0.91 (0.86–0.95)	15	80.0% (63.1–91.6)	88.9% (81.8–94.0)	68.3% (55.7–78.7)	93.7% (88.4–96.7)
Anterolateral	0.91 0.85–0.97)	5	88.6% (73.3–96.8)	82.9% (74.8–89.2)	60.8% (50.6–70.2)	96.0% (90.6–98.4)
Regional						
Anterior	0.84 (0.76–0.92)	2	74.3% (56.7–87.5)	86.3% (78.7–92.0)	61.9% (49.8–72.7)	92.0% (86.4–95.2)
Lateral	0.89 (0.83–0.95)	4	85.7% (69.7–95.2)	80% (72.0–87.1)	56.6% (46.9–65.8)	95.0% (89.3–97.7)
Posterior	0.85 (0.78–0.91)	7	80.0% (63.1–91.6)	76% (67.3–83.5)	49.8% (41.0–59.0)	92.7% (86.7–96.1)

Data is reported as value (95% confidence interval)

*AUROC* area under the receiver operating characteristics curve, *PPV* positive predictive value, *NPV* negative predictive value

**Table 3:** Comparisons between the areas under the ROC using the De Long test

LUS score	Anterolateral	Anterior	Lateral	Posterior
Global	0.903	0.067	0.279	0.016
Anterolateral	–	0.028	0.081	0.093
Anterior	–	–	0.216	0.932
Lateral	–	–	–	0.210
Posterior	–	–	–	–

A *P* value < 0.05 reflects a significant difference

*ROC* receiver operating characteristics curve, *LUS* lung ultrasound

Patients without ARDS had a significant lower global score compared to patients with ARDS (5 [3–9] versus 19 [15–23]; *P* < 0.001]). The posterior score was the main contributor to the global score (Table 4), and was the only score that increased with rising ARDS severity groups (*P* = 0.016, mild vs. severe ARDS). The diagnostic accuracy for ARDS of the posterior scores was comparable to that of the other regional scores, but yielded a lower AUROC than the global score, the lowest specificity, and a clinically irrelevant positive predictive value of 49.8%.

**Table 4:** Detailed LUS scores

LUS score	No ARDS	ARDS		
		Mild	Moderate	Severe
Global	5 (3–9)	16 (11–21)	20 (15–23)	17 (16–25)
Anterior	0 (0–0)	4 (2–5)	4 (1–6)	2 (0–5)
Lateral	1 (0–3)	6 (4–9)	7 (5–8)	6 (6–9)
Posterior	4 (2–6)	7 (5–9)	9 (8–11)	12 (9–12)
*P* value^a^	< 0.001	0.007	< 0.001	0.001

LUS scores are reported as median (interquartile range)

*LUS* lung ultrasound, *ARDS* acute respiratory distress syndrome

^a^Kruskal–Wallis test comparing anterior, lateral and posterior LUS scores. A *P* value < 0.05 reflects a significant difference

### Composite of anterior and lateral scores

Omitting the posterior regions from the global score did not result in a lower diagnostic performance, i.e., the diagnostic accuracy of the anterolateral score was as good as the performance of the global score (AUROC 0.91 [0.85–0.97] vs. 0.91 (0.86–0.95]). Using a cutoff of 5 for the anterolateral score yielded similar predictive values.

## Discussion

The main findings of this study can be summarized as follows: (1) the posterior score is the main contributor to the global score, in patient with ARDS as well as in patients without ARDS; (2) the posterior score, but not the anterior and lateral scores, increases with ARDS severity; however (3), the posterior score has a lower diagnostic accuracy than the global score; and (4) the diagnostic accuracy for ARDS of the simpler anterolateral score is comparable to that of the global score.

This study has several strengths. The study included consecutive invasively ventilated ICU patients, as the ICU where this study was performed follows a clinical protocol dictating that all invasively ventilated patients who are expected to need invasive ventilation > 24 h undergo a LUS examination within the first 2 days of ventilation. Patients included had a wide range of medical and surgical primary diagnoses, and had a prevalence of ARDS comparable to that in other studies [27], increasing its external validity. In addition, each LUS examination followed a strict protocol in which 12 lung regions were scanned, resulting in a detailed representation of all regions, and LUS examinations were performed by a single physician, experienced in performing LUS. The number of regions that were non-examinable was low. The LUS performer remained blinded for clinical data, in particular the presence or absence of ARDS. Finally, ARDS was diagnosed by a team of physicians experienced in using the Berlin Definition [21], which was strictly applied.

The lung aeration scores reported here are very much in line with those from previous investigations [17, 28–30]. Mean global scores in patients with ARDS varied between 18 and 21 in several reports [17, 28, 29], which was confirmed in the present cohort. The previously reported higher global scores with increasing ARDS severity [30] was also found in ARDS patients in the here studied cohort, albeit with a less pronounced linearity.

In line with a previous study, patients without ARDS had a relatively high global score [17]. While aeration was affected in both anterior and posterior regions in the previous study [17], in the present investigation it was the posterior score that contributed most to the global score. This difference may have been caused by the fact that unlike the previous study in which 9 lung points were scanned, here a 12-region approach was used. The noticeable finding that patients without ARDS have relatively high global scores point to the fact that, even in the absence of ARDS, quantifiable dependent lung densities are present in invasively ventilated ICU patients. This is due to the presence of various conditions like hydrostatic lung edema [19], postoperative atelectasis [11], and also infectious infiltrates [20], and do create ‘noise’ that hampers the usefulness of LUS to diagnose ARDS. Therefore, the high optimal cutoff of the global score for diagnosing ARDS, 15 in this representative cohort of patients who are expected to need invasive ventilation for > 24 h, is not surprising.

By omitting the posterior scores, i.e., by combining only the scores for the four anterior and the four lateral regions [8, 10], the abovementioned low signal-to-noise ratio of part of the global score could be overcome. This finding, however, certainly does not mean that posterior regions should not be scanned, as important clinical information could come from the detection or monitoring of pleural effusion, but also assessment of lung inhomogeneity and, e.g., the effects of lung recruitment maneuvers [16]. Also, despite the finding that the posterior score had the lowest diagnostic accuracy for ARDS, this score was the single regional score that showed a significant linear rise with ARDS severity.

A noticeable drop in the global score was found from moderate to severe ARDS, a finding that was mainly driven by an increase in the number of non-dependent regions that were scored as normally aerated or having an ‘A-pattern.’ One possible explanation is that these lung regions were more subject to overdistension, facilitated by the higher levels of PEEP used in these patients. It should be noticed, though, that the number of patients with severe ARDS was rather low. Clearly, this finding deserves more attention in future studies.

The low number of patients with severe ARDS may not necessarily affect the physiological meaning of the results of this study. The modest diagnostic accuracy of posterior LUS scores reflects an unfavorable signal-to-noise ratio in dependent lung regions. This, however, seems mostly due to a higher level of noise, i.e., increased posterior LUS scores in all patients, thus also those without ARDS.

Ventilatory settings, like tidal volume and PEEP, potentially affect LUS scores because they could alter the amount of aerated lung tissue. The effect of the small variations in tidal volume in the current study, though, is negligible, as recently demonstrated [31]. The effects of PEEP-induced recruitment on LUS findings have been described before [12]. Of note, the caregivers within the ICU where this study was performed followed a local guideline that recommended to titrate PEEP to the lowest level at which oxygenation was acceptable, meaning that excessive high PEEP was only used in the most severe cases of ARDS [32]. Interestingly, while the effects of high PEEP on lung aeration could also affect other imaging techniques including chest X-ray and lung CT, this is not accounted for in the currently used Berlin Definition for ARDS [22].

The results of the present study, at least in part, suggest that if LUS scores are used in a heterogeneous process like ARDS, an anterolateral score < 5 has particular interest in excluding ARDS, while posterior scores preserve a specific role in defining ARDS severity, once ARDS is confirmed. This could be of help for clinical reasoning, but is certainly in research purposes.

Several limitations of this study should be acknowledged. As this study took place in one single intensive care unit, its results need external confirmation. The number of patients with severe ARDS was very low, which makes it challenging to draw firm conclusions with respect to this category of patients. Finally, other LUS features, like presence and extent of lung sliding, presence of pleural line abnormalities and subpleural consolidations, and sonographic spared areas, were not collected, while these all may further add to the diagnostic accuracy for ARDS of LUS [33].

## Conclusions

In this cohort of ICU patients expected to need invasive ventilated for > 24 h, the posterior score was the main contributor to the global score, irrespective of the presence of ARDS. While the posterior score had a lower diagnostic accuracy when compared to the global score, it performed best when classifying ARDS severity. Omitting the posterior regions from the global score did not alter the diagnostic accuracy.

## Abbreviations

ARDS: Acute respiratory distress syndrome
AUROC: Area under ROC
ICU: Intensive care unit
LUS: Lung ultrasound
NPV: Negative predictive value
PEEP: Positive end-expiratory pressure
PPV: Positive predictive value
ROC: Receiver operating characteristic curve

## References

[1] Corradi F, Brusasco C, Pelosi P (2014). Chest ultrasound in acute respiratory distress syndrome. Curr Opin Crit Care.

[2] Sweeney RM, McAuley DF (2016). Acute respiratory distress syndrome. Lancet..

[3] See KC, Ong V, Tan YL, Sahagun J, Taculod J (2018). Chest radiography versus lung ultrasound for identification of acute respiratory distress syndrome: a retrospective observational study. Crit Care.

[4] Vukoja M, Riviello E, Gavrilovic S, Adhikari NKJ, Kashyap R, Bhagwanjee S (2014). A survey on critical care resources and practices in low- and middle-income countries. Glob Heart.

[5] Bass CM, Sajed DR, Adedipe AA, West TE (2015). Pulmonary ultrasound and pulse oximetry versus chest radiography and arterial blood gas analysis for the diagnosis of acute respiratory distress syndrome: a pilot study. Crit Care.

[6] Riviello ED, Kiviri W, Twagirumugabe T, Mueller A, Banner-Goodspeed VM, Officer L (2016). Hospital incidence and outcomes of the acute respiratory distress syndrome using the Kigali modification of the Berlin Definition. Am J Respir Crit Care Med.

[7] Lichtenstein D, Goldstein I, Mourgeon E, Cluzel P, Grenier P, Rouby J-J (2004). Comparative diagnostic performances of auscultation, chest radiography, and lung ultrasonography in acute respiratory distress syndrome. Anesthesiology..

[8] Kruisselbrink R, Chan V, Cibinel GA, Abrahamson S, Goffi A (2017). I-AIM (indication, acquisition, interpretation, medical decision-making) framework for point of care lung ultrasound. Anesthesiology..

[9] Vercesi V, Pisani L, van Tongeren PSI, Lagrand WK, Leopold SJ, Huson MMA (2018). External confirmation and exploration of the Kigali modification for diagnosing moderate or severe ARDS. Intensive Care Med.

[10] Volpicelli G, Elbarbary M, Blaivas M, Lichtenstein DA, Mathis G, Kirkpatrick AW (2012). International evidence-based recommendations for point-of-care lung ultrasound. Intensive Care Med.

[11] Monastesse A, Girard F, Massicotte N, Chartrand-Lefebvre C, Girard M (2017). Lung ultrasonography for the assessment of perioperative atelectasis: a pilot feasibility study. Anesth Analg.

[12] Bouhemad B, Brisson H, Le-Guen M, Arbelot C, Lu Q, Rouby J-J (2011). Bedside ultrasound assessment of positive end-expiratory pressure-induced lung recruitment. Am J Respir Crit Care Med.

[13] Enghard P, Rademacher S, Nee J, Hasper D, Engert U, Jörres A (2015). Simplified lung ultrasound protocol shows excellent prediction of extravascular lung water in ventilated intensive care patients. Crit Care.

[14] Bouhemad B, Liu ZH, Arbelot C, Zhang M, Ferarri F, Le-Guen M (2010). Ultrasound assessment of antibiotic-induced pulmonary reaeration in ventilator-associated pneumonia. Crit Care Med.

[15] Rouby J-J, Arbelot C, Gao Y, Zhang M, Lv J, An Y (2018). Training for lung ultrasound score measurement in critically ill patients. Am J Respir Crit Care Med.

[16] Chiumello D, Mongodi S, Algieri I, Vergani GL, Orlando A, Via G (2018). Assessment of lung aeration and recruitment by CT scan and ultrasound in acute respiratory distress syndrome patients. Crit Care Med.

[17] Tierney DM, Boland LL, Overgaard JD, Huelster JS, Jorgenson A, Normington JP (2018). Pulmonary ultrasound scoring system for intubated critically ill patients and its association with clinical metrics and mortality: a prospective cohort study. J Clin Ultrasound.

[18] Yu X, Zhai Z, Zhao Y, Zhu Z, Tong J, Yan J (2016). Performance of lung ultrasound in detecting peri-operative atelectasis after general anesthesia. Ultrasound Med Biol.

[19] Bilotta F, Giudici LD, Zeppa IO, Guerra C, Stazi E, Rosa G (2013). Ultrasound imaging and use of B-lines for functional lung evaluation in neurocritical care. Eur J Anaesthesiol.

[20] Mongodi S, Via G, Girard M, Rouquette I, Misset B, Braschi A (2016). Lung ultrasound for early diagnosis of ventilator-associated pneumonia. Chest..

[21] Ranieri VM, Rubenfeld GD, Thompson BT, Ferguson ND, Caldwell E, The ARDS Definition Task Force (2012). Acute respiratory distress syndrome: the Berlin Definition. JAMA.

[22] Definition Task Force ARDS, Ranieri VM, Rubenfeld GD, Thompson BT, Ferguson ND, Caldwell E (2012). Acute respiratory distress syndrome. JAMA..

[23] Bouhemad B, Mongodi S, Via G, Rouquette I (2015). Ultrasound for “lung monitoring” of ventilated patients. Anesthesiology..

[24] DeLong ER, DeLong DM, Clarke-Pearson DL (1988). Comparing the areas under two or more correlated receiver operating characteristic curves: a nonparametric approach. Biometrics..

[25] Youden WJ (1950). Index for rating diagnostic tests. Cancer..

[26] van Stralen KJ, Stel VS, Reitsma JB, Dekker FW, Zoccali C, Jager KJ (2009). Diagnostic methods I: sensitivity, specificity, and other measures of accuracy. Kidney Int.

[27] Bellani G, Gattinoni L, Van HF, Larsson A, Mcauley DF, Ranieri M (2016). Epidemiology, patterns of care, and mortality for patients with acute respiratory distress syndrome in intensive care units in 50 countries. JAMA..

[28] Haddam M, Zieleskiewicz L, Perbet S, Baldovini A, Guervilly C, Arbelot C (2016). Lung ultrasonography for assessment of oxygenation response to prone position ventilation in ARDS. Intensive Care Med.

[29] Zhao Z, Jiang L, Xi X, Jiang Q, Zhu B, Wang M (2015). Prognostic value of extravascular lung water assessed with lung ultrasound score by chest sonography in patients with acute respiratory distress syndrome. BMC Pulm Med.

[30] Li L, Yang Q, Li L, Guan J, Liu Z, Han J (2015). The value of lung ultrasound score on evaluating clinical severity and prognosis in patients with acute respiratory distress syndrome. Zhonghua Wei Zhong Bing Ji Jiu Yi Xue.

[31] Smit M, Pisani L, Simonis F, Schultz M (2018). Lung aeration as assessed by lung ultrasound is independent of tidal volume size in intensive care unit patients with invasive ventilation. Acute Critical Care European Respiratory Society.

[32] Schultz MJ, de Pont A-C (2011). Prone or PEEP, PEEP and prone. Intensive Care Med.

[33] Copetti R, Soldati G, Copetti P (2008). Chest sonography: a useful tool to differentiate acute cardiogenic pulmonary edema from acute respiratory distress syndrome. Cardiovasc Ultrasound.

